# Chemical composition and evaluation of the antibacterial and Cytotoxic activities of the essential oil from the leaves of *Myracrodruon urundeuva*

**DOI:** 10.1186/s12906-017-1918-6

**Published:** 2017-08-22

**Authors:** Ítalo Diego Rebouças de Araújo, Nayara Coriolano de Aquino, Andreza Conceição Véras de Aguiar Guerra, Renato Ferreira de Almeida Júnior, Renata Mendonça Araújo, Raimundo Fernandes de Araújo Júnior, Kléber Juvenal Silva Farias, José Veríssimo Fernandes, Vânia Sousa Andrade

**Affiliations:** 1 Centro de Biociências (CB), Departamento de Microbiologia e Parasitologia, Universidade Federal do Rio Grande do Norte (UFRN), Natal, RN 59072-970 Brazil; 2 Centro de Ciências, Departamento de Química Orgânica e Inorgânica, Universidade Federal do Ceará (UFC), Fortaleza, CE 60021-940 Brazil; 3 Centro de Biociências (CB), Departamento de Morfologia, Universidade Federal do Rio Grande do Norte (UFRN), Natal, RN 59072-970 Brazil; 4 Centro de Ciências da Saúde (CCS), Departamento de Análises Clínicas e Toxicológicas, Universidade Federal do Rio Grande do Norte (UFRN), Natal, RN 59072-970 Brazil; 5 Centro de Ciências Exatas e da Terra (CCET), Instituto de Química, Universidade Federal do Rio Grande do Norte (UFRN), Natal, RN 59072-970 Brazil; 6 Instituto de Medicina Tropical (IMT), Universidade Federal do Rio Grande do Norte (UFRN), Natal, RN 59072-970 Brazil

**Keywords:** *Myracrodruon urundeuva*, Bactericidal activity, Minimum inhibitory concentration, Chemical characterization, Essential oil, Cytotoxicity

## Abstract

**Background:**

This study evaluated the in vitro activity of essential oil extracted from the leaves of *Myracrodruon urundeuva*.

**Methods:**

The oil was obtained by hydro-distillation and characterized by Gas Chromatography coupled to Mass Spectrometry (GC-MS) and Gas Chromatography with Flame Ionization Detector (GC-FID). The antibacterial activity was evaluated by the broth microdilution technique and the MIF was determined by using growth indicator CTT (2,3,5-triphenyl-tetrazolium) and CBM in BHI agar. The oil’s cytotoxicity was evaluated in HeLa, HEK-293, and Vero E6 cells using MTT, 3- (4,5-dimethylthiazol-2-yl) -2,5-diphenyl tetrazolium.

**Results:**

The oil showed chemical markers, including α-pinene (87.85%), trans-caryophyllene (1.57%), limonene (1.49%) and β -pinene (1.42%), and activity against all strains: *Staphylococcus aureus* (MIC = MBC = 0.22 mg/mL), *Staphylococcus epidermidis* (MIC = 0.11 mg/mL and MBC = 0.22 mg/mL), *Escherichia coli* (MIC = 0.88 mg/mL and MBC = 1.75 mg/mL), *Pseudomonas aeruginosa* (MIC = MBC = 7 mg/mL) and *Salmonella* Enteritidis (MIC = MBC = 0.44 mg/mL). In vitro cytotoxicity tests showed that the oil is not toxic and has slight antitumor activity.

**Conclusions:**

We conclude that the *M. urundeuva* oil results are promising, with prospects of being pharmacologically viable.

## Background

From the earliest times, medicinal plants have been widely used as natural drugs in the treatment, curing and prevention of disease [1]. Among these are bacterial diseases [2–4]. While facing increasing bacterial resistance against routinely used traditional and synthetic antibiotics for the treatment of microbial diseases [5–7], we find that research in plant-based natural products to elucidate new therapies from novel antibacterial agents has grown significantly [8, 9]. Studies report that certain natural antibacterials, when given with standard antibiotics, are even more effective in synergy than the standard drug alone [10–12]. In the cells of resistant pathogens, these naturally occurring molecules supposedly reach different targets than the known antibiotics do.

Among various natural substances with antimicrobial potential, essential oils have been highlighted. These generally consist of a mixture of natural volatile chemical compounds, such as monoterpenes and sesquiterpenes, and their oxygenated derivatives [13]. Such compounds comprise the secondary metabolites of aromatic plants, which are considered to be natural remedies [14, 15] with antimicrobial activity [16–20]. They may therefore be an alternative to overcome the increasing antibacterial resistance of pathogens. Essential oils are also being investigated by some researchers for their anti-cancer potential [21, 22].

Among the aromatic plants whose essential oils are extracted, a promising candidate is an endemic savanna tree, widely distributed in Brazil, that belongs to the family Anacardiaceae: *Myracrodruon urundeuva* (Aroeira of the backwoods) [23]. This plant has been noted for its antimicrobial potential, which was evidenced when molecules obtained from crude extracts of *M. urundeuva* were found to have antibacterial and antifungal properties [24–27]. Additionally, other studies indicate antiparasitic [28], analgesic and anti-inflammatory properties [29], encouraging research on the possible uses for this substance in herbal medicine.

The essential oil extracted from the leaves of *M. urundeuva* comprises a blend of terpenes which have not yet been fully elucidated. Based on these premises, the main objective of this study was to evaluate the in vitro antimicrobial and cytotoxic activities of essential oil extracted from the leaves of *M. urundeuva,* which is typically grown in northeastern Brazil.

## Methods

### Plant material and essential oil extraction

Leaves of *M. urundeuva* were collected from ten (10) specimens grown in the Núcleo de Pesquisa em Agricultura Urbana (NEPAU), Department of Plant Science, Ceará Federal University (UFC), Fortaleza, Ceará, Brazil. The leaves were collected with the aid of pruning shears at four different points in the tree canopy and then were mixed in paper bags to form composite samples. After being cut and mixed, they were packed in a drying oven at 75 °C. The dry weight of the collected plant material was 100 kg. The voucher specimen (No. 48904) was deposited at the Prisco Bezerra Herbarium of the Department of Biology, Ceará Federal University. To extract the essential oil, *M. urundeuva* leaves were hydrodistilled in a Clevenger-type apparatus, as adapted by Gottlieb [30]. In this process, fresh *M. urundeuva* leaves were placed in a 5 L flask, into which 2 L of distilled water were added and the system was boiled for 2 h, until it reached 80 °C, to afford yellowish oils by steam stripping. These were dried over Na_2_SO_4_, stored in sealed glass vials, and preserved under refrigeration before analysis. The yield of the oils (0.02% *w*/w) was calculated from the fresh weight of the plant materials. The oily extract, weighed on a precision scale, revealed a concentration of 0.9 g/mL.

### Identification of essential oil constituents

Essential oils were analyzed by Gas Chromatography coupled to Mass Spectrometry (GC-MS) and Gas Chromatography with a Flame Ionization Detector (GC-FID). The same chromatographic conditions were used for GC-MS (Shimadzu GCMS-QP2010-Plus) and GC-FID (GC-20100FI) analysis. The oil was analyzed using an RTx-5 infused-silica capillary column (30 m × 0,25 mm i.d., 0.25 μm film thickness); the splitless injection used an injector temperature of 220 °C, and the column was programmed to run from 60 to 240 °C at 3 °C/min and held isothermal for 7 min. The flow rate was 1.0 mL min^−1^, and helium and hydrogen (H_2_) were used as the gas carriers for the GC-MS and GC-FID analyses, respectively. The interface temperature of the GC-MS was 240 °C and mass spectra were recorded from 30 to 450 *m/z*, at a scan interval of 0.5 s and an electron impact ionization voltage of 70 eV. Individual components were identified by comparing the acquired mass spectrum with reference data using the Wiley L-Built library and by comparing their retention indices with a commercially available database (NIST 2.0) and the indices described by [31]. Additional identification was obtained by using a compound’s experimental Kovat’s retention index (RI), as calculated from a C9-C24 *n*-alkanes series.

### Microbial isolates

The tested microorganisms were maintained at 8 °C and included *Staphylococcus aureus* (ATCC 25923), *Staphylococcus epidermidis* (ATCC 12228), *Escherichia coli* (ATCC 25922), *Pseudomonas aeruginosa* (ATCC 27853) and *Salmonella* Enteritidis (INCQS 500258). Inoculates were prepared from growth on BHI (Brain Heart Infusion) agar at 37 °C/24 h; Colonies were added in sterile 0.9% saline and adjusted to 0.5 McFarland, which is equivalent to 10^8^ colony-forming units per mL (10^8^ CFU/mL).

### Antibacterial activity

The antibacterial activity of the oil was assessed quantitatively by the broth microdilution technique in 96 well microplates [32]. The test sample (T) was diluted serially at the following concentrations: 450; 225; 112.5; 56; 28; 14; 7; 3.52; 1.76; 0.88; 0.44; 0.22; 0.11; 0.055; 0.028 and 0.014 mg/mL. The positive control (PC) used serial dilutions of gentamicin (Gentamisan - SANTISA®) at a concentration of 0.016 mg/mL or 16 μg/mL. As a negative control (NC), an aqueous solution of 5% Tween 80 was used. We then added 20 μL of the inoculum solution to each of the wells. In the growth control (GC), only the inoculum was added. The microplate was incubated at 37 °C/24 h to determine the minimum inhibitory concentration (MIC). All tests were performed in triplicate.

### Determination of the minimum inhibitory concentration (MIC)

To determine the MIC, 20 μL of CTT (2,3,5-triphenyl-tetrazolium chloride) 0.5% solution was applied to each well to reveal bacterial growth. The MIC readings were performed after incubation at 37 °C for a further 2 h and were defined as the lowest concentrations of essential oil visually inhibiting microbial growth [33].

### Determination of the minimum bactericidal concentration (MBC)

The bactericidal activity of the test oil was measured after reading the MIC by seeding the contents of the micro-titer wells into BHI agar. The most representative wells of the T, PC, NC and GC groups were chosen. From each selected well, 8 μL was seeded in specific regions numbered on the back of the Petri dishes, which were then incubated at 37 °C. After 24 h of incubation, readings were carried out to determine the bactericidal action of the oil. The MBC was considered the lowest concentration that totally prevented microbial growth in BHI agar.

### Cytotoxicity assay

Having shown antibacterial activity, *Myracrodruon urundeuva* essential oil was evaluated for its cytotoxicity to Vero E6 (renal epithelial *Cercopithecus aethiops*), HeLa (human cervical adenocarcinoma) and HEK-293 (human kidney embryonic) cell lines obtained from the Culture Collection of the Federal University of Rio de Janeiro, and cultured in Dulbecco’s modified Eagle’s medium (DMEM) supplemented with 10% fetal bovine serum (FBS), and 1% antibiotic (penicillin/streptomycin). The cells were maintained in an incubator at 37 °C in an atmosphere of 5% CO_2_. To assess the viability and proliferation rate of the cell lines in the presence of the oil, a colorimetric assay based on the tetrazolium salt MTT (3- (4,5-dimethylthiazol-2-yl) -2,5-diphenyltetrazolium bromide) (Sigma-Aldrich, Germany) was performed as described by Mosmann (1983) [34]. This technique evaluates the metabolic activity of the cells by quantifying the MTT reduction by NADPH and NADH-associated dehydrogenases that results in the production of formazan crystals within the cells, giving the characteristic coloration to the medium. For this study, cells were placed in 96 well plates with a density of 5 × 10^3^ cells/well. After 24 h under the culture conditions, serum deprivation was performed, and, after another 24 h, we applied *M. urundeuva* essential oil at concentrations of 0.275 mg/mL, 0.55 mg/mL, 1.1 mg/mL, 2.2 mg/mL and 4.4 mg/mL to the cell strains. After the cells were treated for 24 h or 48 h, 100 μL/​​well of MTT (5 mg/mL) was added and incubated for 4 h. After this, the medium was removed and 100 μL of ethanol was added to each well. The plates were shaken and the absorbance obtained in a microplate reader (Epoch - BioTek Instruments Inc., USA) at 570 nm, using Gen5 Data Analysis version 2.0 software (BioTek Instruments Inc., USA). The growth control consisted only of cell culture in the culture medium. The assays were assessed in triplicate and the cell viability percentage was calculated as follows:

1 $$ CV\%=\left( Abs\  sample/ Abs\  growth control\right)\ x100 $$

### Statistical analysis

To compare the cell viability of the Vero E6, HeLa and HEK-293 cells lines between 24 h and 48 h, a Wilcoxon test (R-3.1.1 Software) was performed on the data obtained from the above formula.

## Results

### Chemical characterization of the essential oil of *M. urundeuva*

During the chemical characterization of *M. urundeuva* leaf essential oil, the presence of four major chemical markers was detected with intraspecific variability: *α*-pinene (87.85%) as the major constituent, *trans*-caryophyllene (1.57%), limonene (1.49%) and *β*-pinene (1.42%). The constituents were identified by mass spectrometry (GC-MS and GC/FID), shown in Table 1.

**Table 1:** Essential oil components identified by GC-MS and GC/FID

#	Compound	^a^RI	^b^RIc	Essential Oil (%)
1	*α*-Pinene	939	958	87.85
2	Canfene	946	964	0.28
3	*β-*Pinene	979	982	1.42
4	Myrcene	990	991	1.82
5	δ-3-Carene	1011	1006	0.22
6	Limonene	1029	1022	1.49
7	Eucalyptol	1031	1024	0.43
8	Terpinolene	1088	1074	0.43
9	*trans*-Cariofilene	1419	1419	1.57
10	Aromadendrene	1441	1438	0.57
11	Viridiflorene	1496	1495	0.18
12	Viridiflorol	1592	1582	0.63
	Hydrocarbons monoterpenes			93.51
	oxygenated monoterpenes			0.43
	hydrocarbons sesquiterpenes			2.32
	Oxygenated sesquiterpenes			0.63
	Total identified			96.89

^a^RI: Kovat’s retention index

^b^RIc: Kovats Indices calculated through the equation Ric = 24.07Tr + 818

### MIC and MBC determination

The MIC and MBC results are shown in Table 2. The controls validated the tests. The positive control (PC) showed no bacterial growth at higher concentrations, demonstrating the effectiveness of gentamicin as a positive control in the test, since the antibiotic’s inhibitory effect on the bacteria occurred up to a concentration of 0.004 mg/mL (4 μg/mL), with equivalence between the MIC and MBC. Bacterial growth occurred in all assays for both the negative control (NC) and for the growth control (GC), confirming the non-bacterial inhibition of Tween 80 and the viability of the strains used and demonstrating the antibacterial effect of *M. urundeuva* oil.

**Table 2:** Minimum Inhibitory Concentration (MIC) and Minimum Bactericidal Concentration (MBC) of the *Myracrodruon urundeuva* essential oil

	Concentrations (mg/mL)			
	*M. urundeuva* essential oil		Gentamicin	
Bacteria	MIC	MBC	MIC	MBC
*Staphylococcus aureus* (ATCC 25923)	0.220	0.220	0.004	0.004
*Staphylococcus epidermidis* (ATCC 12228)	0.110	0.220	0.004	0.004
*Escherichia coli* (ATCC 25922)	0.880	1.750	0.004	0.004
*Pseudomonas aeruginosa* (ATCC 27853)	7.000	7.000	0.004	0.004
*Salmonella* Enteritidis (INCQS 500258)	0.440	0.440	0.004	0.004

*ATCC* American Type Culture Collection, *INCQS* Instituto Nacional de Controle de Qualidade em Saúde (Fiocruz - Brazil)

### Cell-based toxicity results

**Fig. 1:**
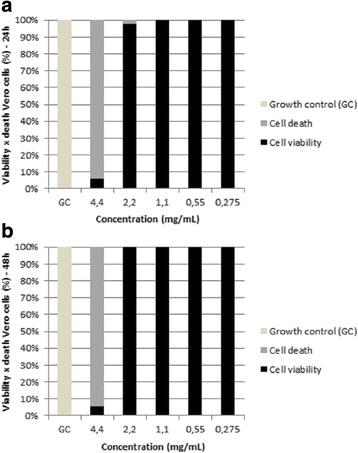
Percentages Vero cell viability. Treated with differing concentrations of *M. urundeuva* essential oil after 24 h (**a**) and after 48 h (**b**). GC: growth control

In the cell viability assay for the Vero E6 line, it was shown in the first reading after 24 h of exposure that there was toxic activity at concentrations of 4.4 mg/mL and 2.2 mg/mL of oil, inducing cell death at 93.91% and 2.32%, respectively. However, the lower oil concentrations of 1.1 mg/ml, 0.55 mg/ml, and 0.275 mg/ml did not affect the viability of the cells. In the second reading, after 48 h of exposure, cell toxicity occurred only at the highest concentration, 4.4 mg/mL, which killed 94.26% of cells. For the other concentrations of 2.2 mg/mL, 1.1 mg/mL, 0.55 mg/mL and 0.275 mg/mL, cell viability was not affected by the oil. However, statistically, there was no significant difference between the cellular viability values at 24 h and 48 h (*p* = 0.7972 (*p* > 0.05), indicating that the oil only inhibits Vero E6 cells at the highest concentration, 4.4 mg/mL. The results are shown in Fig. 1.

**Fig. 2:**
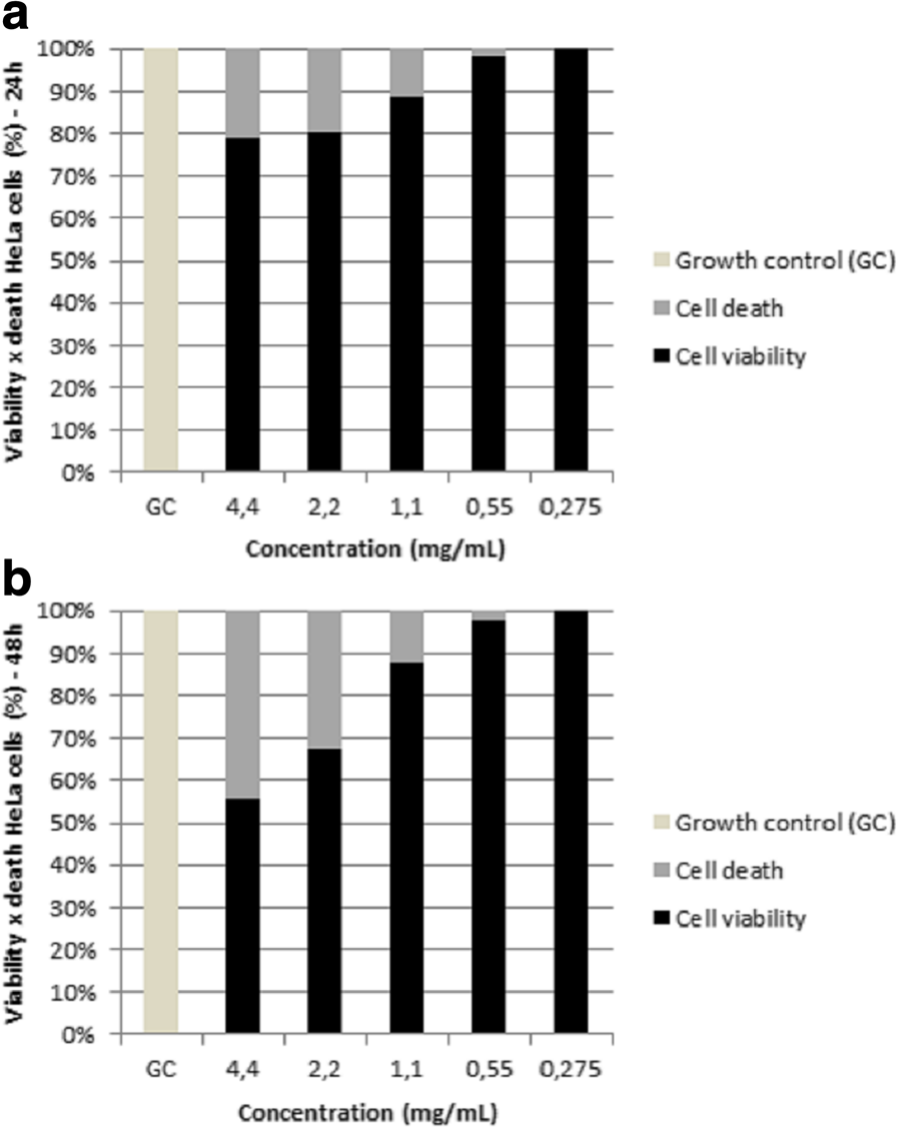
HeLa cell viability. Treated with different concentrations of *M. urundeuva* essential oil for 24 h (**a**) and 48 h (**b**). GC: growth control

After treating HeLa strain for 24 h, we observed that the largest *M. urundeuva* oil concentrations had a greater inhibitory effect on tumor cell proliferation. Accordingly, the oil showed inhibitions from 11% to 21% between the doses of 1.1 mg/mL and 4.4 mg/mL. After treatment for 48 h, concentrations of 2.2 mg/mL and 4.4 mg/mL showed a greater inhibition of proliferation – 32.4% and 44.3%, respectively. This suggests that, although there was no statistically significant difference between the viabilities observed after 24 h and 48 h (*p* = 0.8085; *p* > 0.05), the oil had notable antitumor activity after 24 h that intensified over longer exposure times, as shown in the 48 h reading, which slightly decreased cell viability at all concentrations (Fig. 2).

**Fig. 3:**
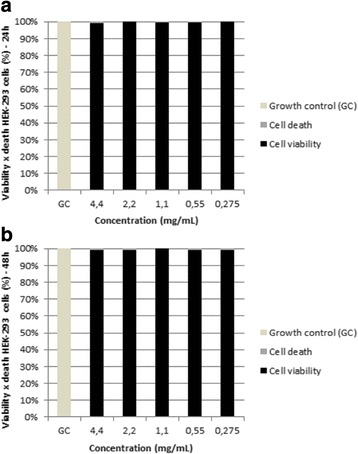
HEK-293 cell viability. Treated with different concentrations of *M. urundeuva* essential oil for 24 h (**a**) and 48 h (**b**). GC: growth control

For the non-tumor cell strain HEK-293, we observed that the oil exerted no toxic effects. At 24 h, cell proliferation occurred at levels near or above those of the untreated control cells. At 48 h we observed the same result. The statistical analysis showed that there was no difference between cell viability percentage in 24 h and 48 h, and these were statistically the same, since the *p* value obtained from the Wilcoxon test was *p* = 1 (*p* > 0.05) (Fig. 3).

## Discussion

Little has been reported on the antibacterial activity of *Myracrodruon urundeuva* essential oil [27]. However, studies of other essential oils from the same plant (with different chemical compositions) have been tested against some of the bacteria herein tested. Among these, *S. aureus* [26, 35–37], *E. coli* [26, 36, 37], and *P. aeruginosa* [37], were inhibited by such extracts, showing the antimicrobial activity of *M. urundeuva*.

However, in this study, the *M. urundeuva* leaf essential oil showed antibacterial activity against all tested strains. Such activity is probably attributable to the terpenoids, since they represented a higher proportion of the constituents in the GC-MS and GC/FID analyses. Similarly, other studies attribute antimicrobial activity to these secondary metabolites due to being abundant constituents in these essential oils [16–20, 27, 28, 38].

This study presents α-pinene (87.85%) as the major constituent in *M. urundeuva* leaf essential oil. However, it is not known whether α-pinene is chiefly responsible for the bactericidal action of the oil, or if one of the other terpenes promotes such action, since in other studies, the essential oils from *M. urundeuva* leaves have shown different terpene proportions from our results [27, 28, 39, 40]. It is important to consider differences in the chemical composition of essential oils extracted from plants of the same species. These differences arise from factors such as geographic variation, climate, soil, planting modes, fertilizers used, time of collection, and the post collection processing and extraction techniques employed [41–44]. It is believed that the *M. urundeuva* essential oil’s antibacterial activity is likely conferred by terpenes, since they are abundant in the composition.

Some papers support the hypothesis that α-pinene is responsible for the antimicrobial action reported in the present study. In work by Leite et al. [45], this terpene was isolated and showed antibacterial activity against *S. aureus* and *S. epidermidis*. Other studies that identified α-pinene as a major constituent in the composition of essential oils from plants other than *M. urundeuva* also observed antibacterial activity against Gram-negative and Gram-positive bacteria [46–49].

In the current study, we observed *M. urundeuva* essential oil’s bactericidal action for all of the bacteria tested: Gram-positive (*S. aureus* and *S. epidermidis*) and Gram-negative (*E. coli, P. aeruginosa* and *S. enteritidis*). The MIC and MBC data show higher antibacterial activity against Gram-positive bacteria than Gram-negative bacteria. This is probably due to the greater complexity of the Gram-negative cell wall [50], which interferes with any direct action on the pathogen metabolism. Among the five strains tested, *S. epidermidis* (MIC = 0.11 mg/mL) was found to be the most sensitive to *M. urundeuva* essential oil, showing even higher sensitivity than the other Gram positive bacteria, *S. aureus* (MIC = 0.22 mg/mL). For the three Gram-negative bacteria, *M. urundeuva* essential oil more strongly inhibited *S. enteritidis* (MIC = 0.44 mg/mL) compared to *E. coli* (MIC = 0.88 mg/mL) and *P. aeruginosa* (MIC = 7.0 mg/mL). *P. aeruginosa* was the least sensitive to the essential oil, possibly due to the ability of *P. aeruginosa* to produce biofilm [51], which further complicates direct action of the aromatic oil component against the bacterial cell.

In addition to *M. urundeuva*, the literature reports studies that prove the effectiveness of essential oils obtained from other plants [52–59]. Although these other plants show similar or higher antibacterial activities than *M. urundeuva*, one should not underestimate its promising antibacterial effect.

However, some essential oils extracted from other plants exert weaker antimicrobial activity against the bacteria tested herein because of weaker inhibition of bacterial growth, higher MIC values, or a demonstrated bacterial resistance [60–68]. These results, when compared with those obtained in the present study, reveal the more promising antibacterial power of *M. urundeuva* essential oil, likely because it contains specific active components.

For HeLa cells, *M. urundeuva* oil shows increasing cytotoxicity over longer exposure times, indicating possible anticancer activity. For the Vero E6 strain, *M. urundeuva* oil cytotoxicity decreased with increasing exposure time, i.e., the greater the exposure time the lower the cytotoxic activity, and the higher the cell viability. This effect was also observed for the HEK-293 line, where cell viability was superior to both Vero strain readings. For the Vero E6 and HEK-293 strains, these results confirm that the essential oil is potentially non-cytotoxic to normal human and mammalian cells in general.

Literature data on *M. urundeuva* essential oil cellular toxicity are still scarce. However, some researchers have evaluated the toxicity of *M. urundeuva* oil, stimulating further research on their use as a source of medicinal products. In the study by Carvalho et al. [28] its essential oil showed low cytotoxicity for human erythrocytes. Work by Ferreira et al. [69] highlights the antiproliferative power of the methanolic extract obtained from *M. urundeuva* against HL-60 lines (leukemia) cancer, SF-295 (glioblastoma), HCT-8 (colon), and MDA/MB-435 (melanoma) cells, indicating the possible antitumor effects of *M. urundeuva* extracts.

Despite the data obtained from these cell toxicity tests, additional in vitro assays are needed and will be conducted in the future, such as cytotoxicity against normal human cells originating from other tissues, and further investigation of its antitumor activity in fibroblasts and other neoplastic cells. Further testing is needed to prove the effectiveness of the oil in vivo so it can be considered pharmacologically viable. Finally, *M. urundeuva* oil showed significant antibacterial activity, notable antitumor effectiveness, and very low cell toxicity.

## Conclusion

Essential oil extracted from the leaves of *Myracrodruon urundeuva* showed promising Gram-positive and Gram-negative antibacterial activity. Additionally, the in vitro cytotoxicity test results were satisfactory, since the oil was not toxic to human cells and displayed an antitumor effect, showing its potential as a reliable source for phyto-pharmaceutics in the future. Although further in vivo assays and in vitro toxicity studies are necessary in normal human cells to prove its efficacy and safety in pre-clinical use, the results obtained here for *M. urundeuva* essential oil are already promising from a pharmacological point of view and suggest future applications.

## Abbreviations

ATCC: American Type Culture Collection
BHI: Brain heart infusion
CFU: Colony-forming units
CTT: 2.3.5-triphenyl-tetrazolium chloride
DMEM: Dulbecco’s modified Eagle’s medium
FBS: Fetal bovine serum
GC: Growth control
GC-FID: Gas Chromatography-Flame Ionization Detector
GC-MS: Gas chromatography-mass spectrometry
INCQS: Instituto Nacional de Controle de Qualidade em Saúde
MBC: Minimum Bactericidal Concentration
MIC: Minimum Inhibitory Concentration
MTT: [3-(4.5-dimethylthiazolyl)-2.5-diphenyl-tetrazolium bromide]
NC: Negative control
PC: Positive control
RI: Kovat’s retention index
